# Coupled Motion of Contact Line on Nanoscale Chemically Heterogeneous Surfaces for Improved Bubble Dynamics in Boiling

**DOI:** 10.1038/s41598-017-16035-8

**Published:** 2017-11-16

**Authors:** Arvind Jaikumar, Satish G. Kandlikar

**Affiliations:** 1 Microsystems Engineering, Rochester Institute of Technology, 76 Lomb Memorial Drive, Rochester, NY 14623 USA; 2 Mechanical Engineering, Rochester Institute of Technology, 76 Lomb Memorial Drive, Rochester, NY 14623 USA

## Abstract

We demonstrate that the contact line (CL) motion on energetically heterogeneous solid surfaces occurs in a coupled fashion as against the traditional staggered stick-slip motion. Introducing chemical inhomogeneities at nanoscale induces a local change in dynamic contact angles which manifests as a smooth and continuous motion of the CL. Nanoscale chemically inhomogeneous surfaces comprising of gold, palladium and nickel were generated on copper substrates to demonstrate the underlying CL dynamics. The spatial variations of chemical constituents were mapped using elemental display scanning electron microscope images. Further, the coupled and stick-slip motion was confirmed for a sliding water droplet on these surfaces, and then used in studying the pool boiling bubble dynamics of a single bubble from nucleation to departure. The coupled motion was seen to increase the CL velocity thereby increasing the contribution from transient conduction heat transfer. Consequently, a ~2X increase in the boiling critical heat flux (CHF) was observed. Enhancing the pool boiling performance by introducing nanoscale surface features is an attractive approach in many applications and this work provides a framework and understanding of the CL motion induced through the chemical inhomogeneity effects.

## Introduction

Interface motion and its dynamics are at the heart of numerous wetting and non-wetting applications in microfluidics, heat transfer, ink-based printing, and purification industry. Solid surfaces commonly employed in these applications are inherently rough due to surface features as a consequence of the manufacturing process or energetically heterogeneous due to a change in chemical composition. The roughness implicates a change in the contact angle measured along different viewing directions while the heterogeneity manifests through the motion of the contact line (CL)^1^. For surfaces with roughness features, the contact angle (and hysteresis) may take a predictable character. Consequently, the motion of the CL occurs in a stick-slip fashion when the liquid interface is advanced or receded^2,3^. The Wenzel equation successfully predicts the expected contact angles for roughened surfaces by applying a *rugosity* factor. This factor is computed as the surface area normalized over a smooth surface without roughness features, thereby providing a strategy to increase or decrease wettability of a solid surface through roughness modulation.

The wetting behavior in the chemical heterogeneity case is generally ignored since the scale of the heterogeneous irregularities is often isolated from the feature sizes and the interline at the solid-liquid-vapor/gas interface is affected only in the microscale. However, in applications that employ nanoscale coatings, the motion of the CL due to the heterogeneity induced by the chemical constituents has a significant bearing on the wetting behavior^4^. In this case, the Cassie-Baxter equation^5^ may be employed in estimating the apparent CA through the known area fractions of the two constituents on the surface. Such surfaces are beneficial in driving the CL through contact angle manipulation as it traverses through different chemically constituted nanoscale patches.

Brochard^6^ demonstrated the motion of the CL towards lower surface energy on a surface treated with chemical gradients and towards higher surface energies on thermally induced gradients. Decker and Garoff^7^ experimentally investigated the effects of contact angle hysteresis and CL motion on chemically heterogeneous surfaces. They demonstrated that chemical change induces a specific change in the advancing and receding angles, but not necessarily in the contact angle hysteresis. Additionally, they showed that the spatial variation of wettability influences the CL motion on the surface. Varagnolo *et al*.^8^ showed that large wettability contrasts between hydrophobic and hydrophilic patterns on a surface reduces the effective CL speed by causing the interface to undergo a stick-slip motion. These studies indicate that the local wettability change on chemically inhomogeneous surfaces drives the CL motion.

## Hypothesis

Tanner’s law (Eqn. 2) describes the dependence of the hydrodynamic factors influencing liquid travel with the dynamic contact angle. In this context, the capillary number as a function of viscous (*μ*), surface tension (*σ*) and CL velocity (*V*) is used to represent the hydrodynamic effects as:

1 $$Ca=\frac{V\mu }{\sigma }$$

which is related to the dynamic contact angle,

2 $${\theta }_{D}^{3}=(const)Ca$$

A manifestation of this effect can be translated to surfaces with nanoscale chemical inhomogeneity or surfaces comprising of nanoscale area fractions of different chemical constituents. On these surfaces, the local change in dynamic contact angles along the CL due to variation in chemical composition is capable of altering the stick-slip motion. This altered CL motion is hypothesized to increase the CL velocity in accordance with the Tanner’s law (Eqn. 2). On a surface lacking chemical inhomogeneities, the typical stick-shift motion is expected to adversely affect the CL velocity due to increased pinning effects.

**Figure 1:**
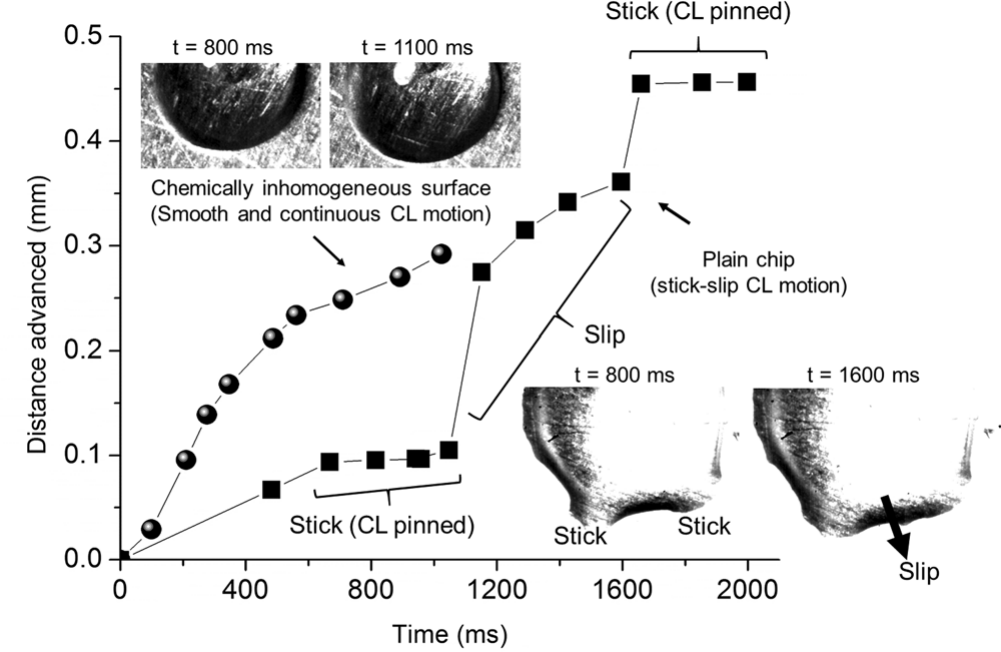
Distance advanced by the liquid over time for (**a**) plain chip showing stick-slip motion, and (**b**) chemically inhomogeneous surface (gold and palladium) showing coupled CL motion. (Insets) High speed images of the CL motion obtained using a Photron fastcam at 4000 fps.

An experimental setup was devised to validate the hypothesis using contact angle goniometer, distilled water and a high speed camera. A plain copper test section was used as the control while another copper test section sputter-coated with gold and palladium served as the surface comprising of chemical inhomogeneities. The surfaces were held at an angle on a leveled stage for the droplets to slide. A motor controlled system was employed to inject 5 µl liquid droplet on the surface while simultaneously capturing high speed videos at 4000 fps. Figure 1 shows the measured distance of the advancing liquid front and its corresponding CL motion profile for the plain and chemically inhomogeneous surfaces. In the case of a plain surface, the CL motion reveals a clear pinning event, and subsequent interface motion (slip) between the pinning events. This is further confirmed by the high speed images shown in the figure as insets. Consequently, the CL advances in a non-continuous or staggered motion. The chemically inhomogeneous surface reveals a smooth motion of the CL as confirmed by the measured distance and high speed images. This smooth and continuous motion was termed as coupled jumps by Decker and Garof^7^. Following their terminology, this smooth and continuous interface motion on nanoscale inhomogeneous surfaces is referred to as the coupled CL motion. The change in dynamic contact angles along the CL due to chemical inhomogeneities present on the surface drives the CL motion.

The CL speed^9^, contact angle^10^ and the nature of its motion are of significant interest in boiling heat transfer. Continual demand for higher system efficiency to reduce cost, improve reliability and increase safety drives methods to enhance the boiling performance characteristics^11–13^. At the heart of any boiling system is a single bubble which undergoes nucleation, growth and departure^14^. During this cycle, referred to as the ebullition cycle, heat is transferred through microconvection, microlayer evaporation, and transient conduction mechanisms^15,16^. The rapid motion of the liquid-vapor interface disturbs the thermal boundary layer in its immediate vicinity which induces microconvection enhancement^17^. Microlayer evaporation occurs when a thin liquid film trapped underneath a growing bubble aids in evaporation promoting bubble growth^18^. The transient conduction occurs as the bubble interface retracts, causing the advancing liquid to sweep over the heater surface at the bubble base^16^. Exploitation of these underlying mechanisms through micro/nanoscale surface enhancements can deliver remarkable improvements in the performance limits. The addition of surface structures has been reported to provide a variety of enhancement levels. A summary of these enhancements is provided in Table 1.

**Table 1:** Summary of multiscale pool boiling enhancement features and mechanisms.

Surface	Enhancement scale	Heat transfer mechanism
Nucleating regions with feeder channels^19^	100–500 µm	Separate liquid-vapor pathways through bubble induced macroconvection
Porous coatings^20^	30–200 µm	Increased nucleation activity and evaporation through wick microstructures
Microgrooves/ridges^21^	10–50 µm	Evaporation through microlayer fragmentation
Hierarchical wicking^22^	8–32 µm	Improved capillary wicking increases thin film evaporation contribution
Hierarchical roughness^23^	5–12 µm	Increasing effective CL length with stick-slip interface motion
Bi-philic surfaces^24^	5 nm in height, covering >10–100 µm^2^ area	Wettability patterns improves the nucleation and wetting characteristics

The heat transfer due to the CL motion is significantly higher than the bulk heat transfer as indicated in multiple reports^25,26^. The absence of stick-slip motion on chemically inhomogeneous surface formed the basis of designing surfaces to improve the CL heat transfer. A chemical inhomogeneity is introduced on the surface due to compositional gradients during the deposition process. These inhomogeneities are typically neglected in microscale features, however their effects are more pronounced in the CL region as demonstrated here. This study provides a framework to enhance boiling heat transfer at the nanoscale by improving the CL characteristics.

## Preparation of Samples

Plain and nanoscale chemically heterogeneous copper chips, 17 mm square were used as the test sections (see Figure S4b in supporting information) similar to Jaikumar and Kandlikar^27^ in the current study. The chemical coatings were applied on a central area of 1 cm^2^. This area was polished to achieve a smooth surface finish. In addition to polishing, a careful cleaning process with isopropyl alcohol and subsequent rinsing with deionized water was done on all the test chips. The measured arithmetic mean roughness on the plain chip was 1.1 ± 0.2 µm.

**Figure 2:**
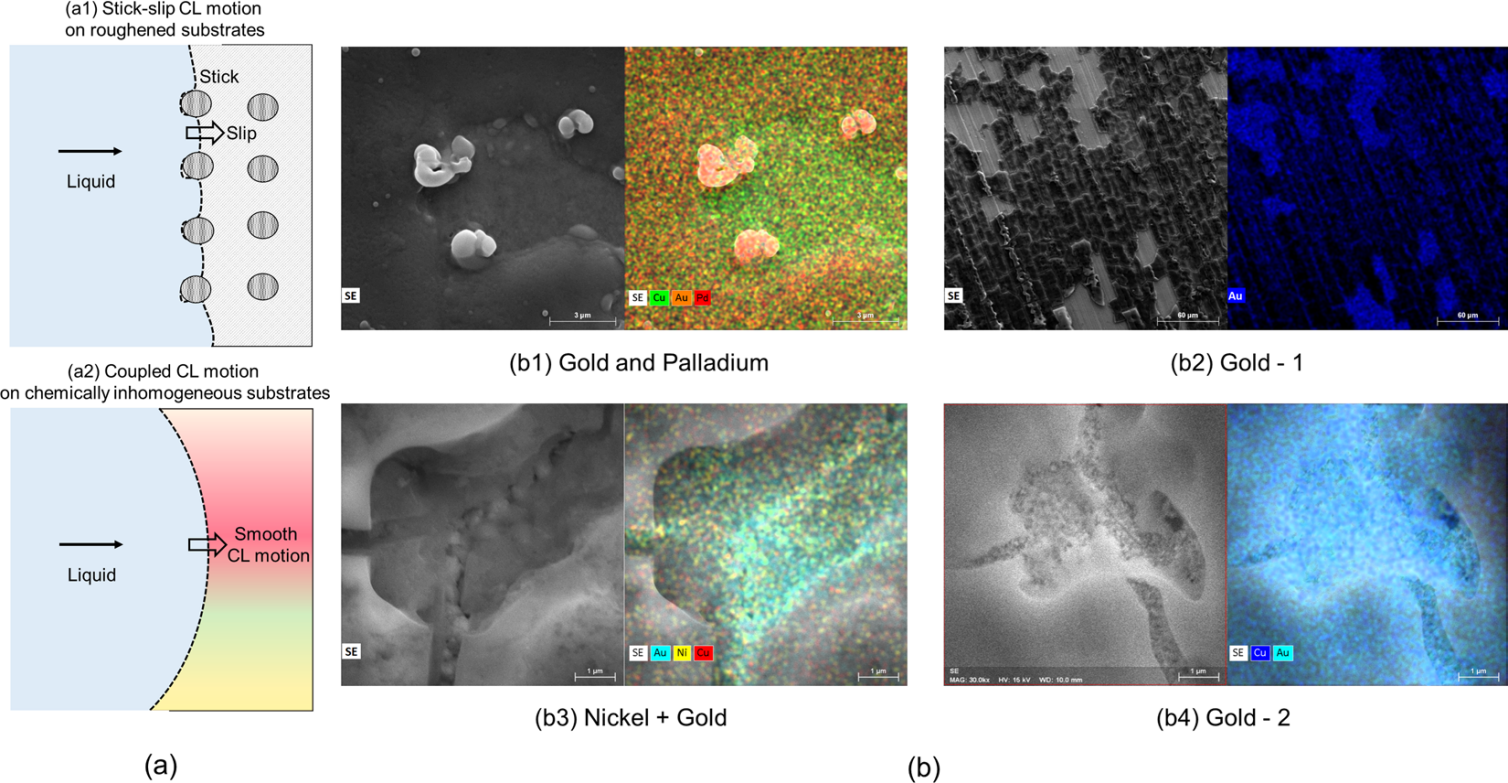
(**a**) Schematic representation of stick-slip motion (a1) and coupled CL motion (a2). (**b**) Scanning electron microscope and elemental display mapping images on gold and palladium (b1), gold-1(b2), nickel and gold (b3), gold-2 (b4) samples. The images show the existence of a compositional change which drives the CL motion on the surface.

Sputter coating and electroplating techniques were devised to deposit the desired coatings on the surface. For sputter coating, a solution comprising of gold and palladium was evenly sputtered on the surface in an argon atmosphere. An electroplating process was employed to deposit layers of gold and nickel on the surface. Gold-1 and gold-2 surfaces were generated by varying the number of electrochemical strokes (see methods section). Figure 2(a1 and a2) shows the schematic representation of staggered and continuous CL motions with the plain and chemically inhomogeneous surfaces, respectively. Figure 2b shows the scanning electron microscope and elemental display images on the prepared samples. The chemical inhomogeneity due to compositional changes (b1 and b3), imperfect surface coverage (b2) and defect-induced changes (b4) were captured in these images.

The advancing and receding contact angles were measured during the bubble cycle time using high speed images obtained during the boiling tests. The arithmetic mean roughness of the chemically enhanced coatings was obtained through a Keyence® laser confocal microscope and is summarized in Table 2. The repeatability of the contact angle and roughness measurements was confirmed by conducting 10 measurements on each test surface. The values show good agreement with maximum standard deviation in contact angle and roughness being ±2° and ±0.5 µm, respectively. In addition to the contact angle and roughness measurements, wickability tests were conducted on the samples using a video contact angle goniometer. In all the tests conducted here the droplet attained its static contact angle, without showing any further spreading behavior.

**Table 2:** Contact angle and roughness values on the fabricated samples.

Surface	Contact angle (deg.)		Roughness, µm
	Advancing	Receding	
Plain chip	67.5	40.2	1.1
Gold and palladium	59.5	39.9	0.94
Gold-1	45	30.2	1.34
Gold-2	44.3	41.7	0.87
Nickel and gold	50.5	40.4	0.93

The contact angles were determined from high speed images obtained at 4000 fps using a Photron fastcam camera. The arithmetic mean roughness (R_a_) was obtained using a Keyence laser scanning microscope using a 10X magnification lens.

## Role of Heat Transfer Mechanisms

Fundamental heat transfer mechanisms in boiling centers around rewetting mechanisms to improve the nucleation and heat transfer characteristics^28,29^. Primary enhancements that have been identified to affect the CL dynamics are the roughness modification by increasing the CL length and liquid spreading through increased wicking. The lack of microscale roughness features and wicking (spreading) behavior indicates that other mechanisms were responsible for the enhancement in the tested samples.

The complex and chaotic nature of bubbles at higher heat fluxes impedes the ability to visualize close to the heater surface. Therefore, several earlier experimental and theoretical studies have based their work on explaining the observed mechanisms using visualization at lower heat fluxes, and hypothesizing similar liquid-vapor transport to occur at higher heat fluxes^12,27^. Another approach undertaken by researchers is to capture a single bubble from nucleation to departure on the heater surface through which the heat transfer mechanisms can be analyzed^15,16^. During this process, heat transfer can be effectively partitioned as from microlayer evaporation and transient conduction mechanisms. To this effect, an isolated individual nucleating bubble in saturated water was analyzed through high speed imaging. A photron fastcam was used to capture videos at 4000 fps. A thorough calibration procedure was undertaken prior to capturing the videos.

**Figure 3:**
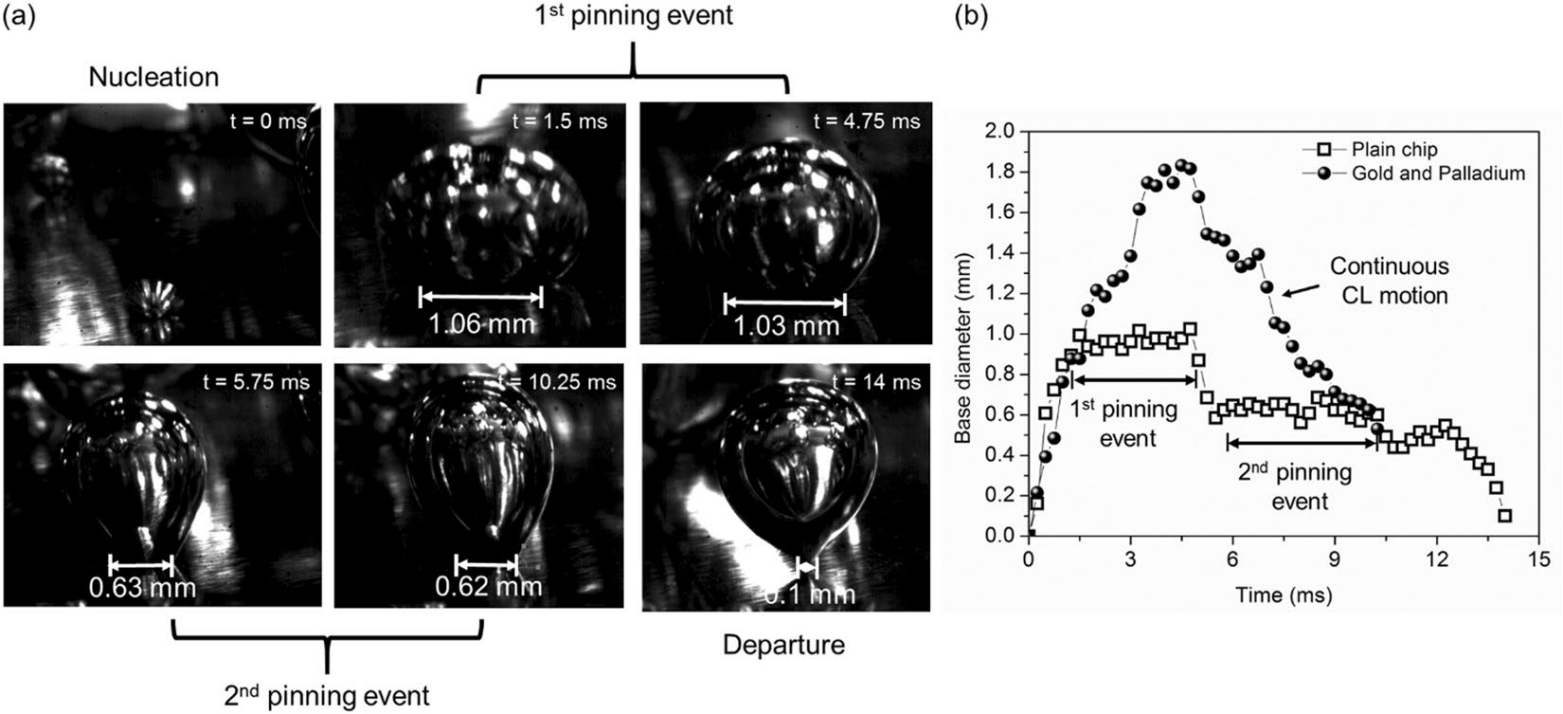
(**a**) High speed image sequence of a single bubble from nucleation to departure. This image sequence shows the two pinning events encountered by the bubble during its advancing motion. (**b**) Measured bubble base diameter motion for a plain chip and a chemically enhanced surface.

Figure 3a shows the high-speed image sequence obtained on a plain surface under saturated boiling conditions. Characteristic CL motion can be identified for the plain chip and the chemically enhanced surface (see Figure S1 in supporting information for high speed images) in accordance with the hypothesis described previously. The image sequence shows that a bubble nucleating on a plain chip undergoes two pinning events during its advancing motion. The motion of the CL occurs in a typical stick-slip fashion. In this motion, the CL remains pinned at defects, while the interface between the pinning sites advances the CL due to built-up inertia. This type of pinning event was seen to be absent on the chemically enhanced surface. The bubble base diameter measured on the plain chip was compared with that obtained on a chemically enhanced surface (Fig. 3b) which shows a continuous CL motion. The CL in such an arrangement seems to occur smoothly which is referred to as a coupled motion (without stick-slip) in this work.

**Figure 4:**
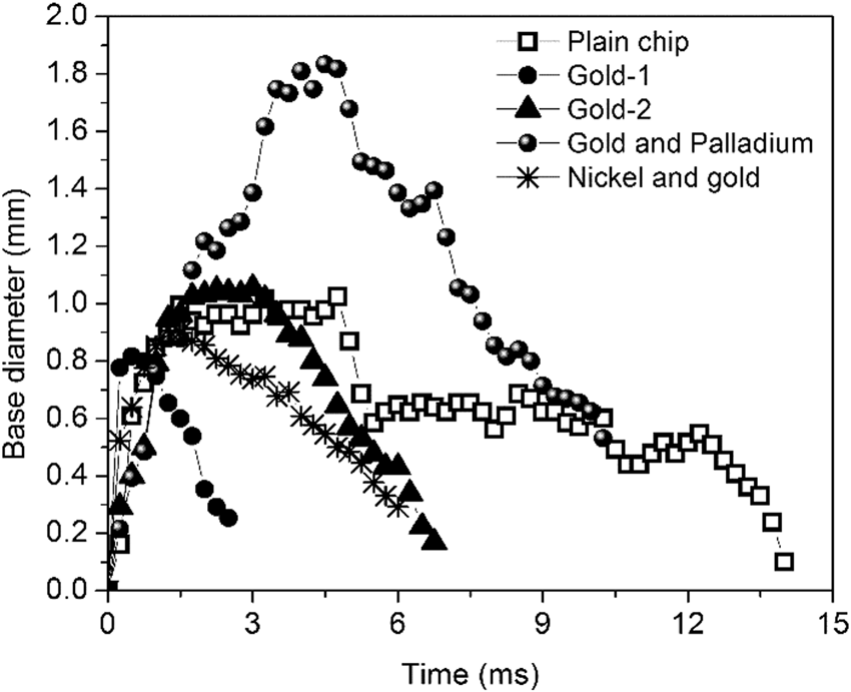
Bubble base diameter from nucleation to departure for the samples investigated in this study. The bubble base radius was obtained after a calibration procedure before capturing the high-speed images. The initial motion till a surface attains maximum base diameter is the receding motion (or bubble growing), while the latter is the advancing (or bubble detaching) motion.

The base diameter of each bubble on all the surfaces was measured over its cycle time at a heat flux of ~20 W/cm^2^. To ensure repeatable data, four isolated bubbles were analyzed on each surface (Figure S2 in supporting information) and showed good agreement with each other. Figure 4 shows the measured diameter for the plain chip and the chemically enhanced surfaces. Several key observations were derived from this figure. Firstly, the CL motion on the plain surface revealed a staggered motion during its initial receding and subsequent advancing motion. This suggested that the CL motion suffers pinning and de-pinning (moving) events during its cycle time. Secondly, the CL motion on the chemically enhanced surface shows a continuous (coupled) motion lacking any pinning events. This effect was attributed to the presence of chemical constituents on the surface. Thirdly, the maximum base diameter observed for each surface is an important consideration for the microlayer heat transfer contribution.

Microlayer evaporation occurs when a thin liquid film is trapped under a growing bubble. Evaporation of this thin film leads to a substantial increase in the bubble growth rate^21^. The total energy from the microlayer can be estimated by analyzing (i) the heat transfer from the wall during the microlayer event, and (ii) the initial sensible energy of the microlayer^15^. The thickness of the microlayer contributes to the total heat transferred during the process. Since, the microlayer thickness typically measured are in the microscale (less than 10 µm), the temperature profile within this layer is expected to be linear. The total microlayer heat transferred over the bubble cycle time can be estimated using

3 $${\dot{Q}}_{ME}=\frac{{\rho }_{l}{V}_{\delta o}{h}_{lv}}{t}$$

where, ${\dot{Q}}_{ME}$ is the heat transferred from the microlayer, ${\rho }_{l}$, ${V}_{\delta o}$, ${h}_{lv}$ and *t* are the liquid density, volume of the microlayer, latent heat and time, respectively.

**Figure 5:**
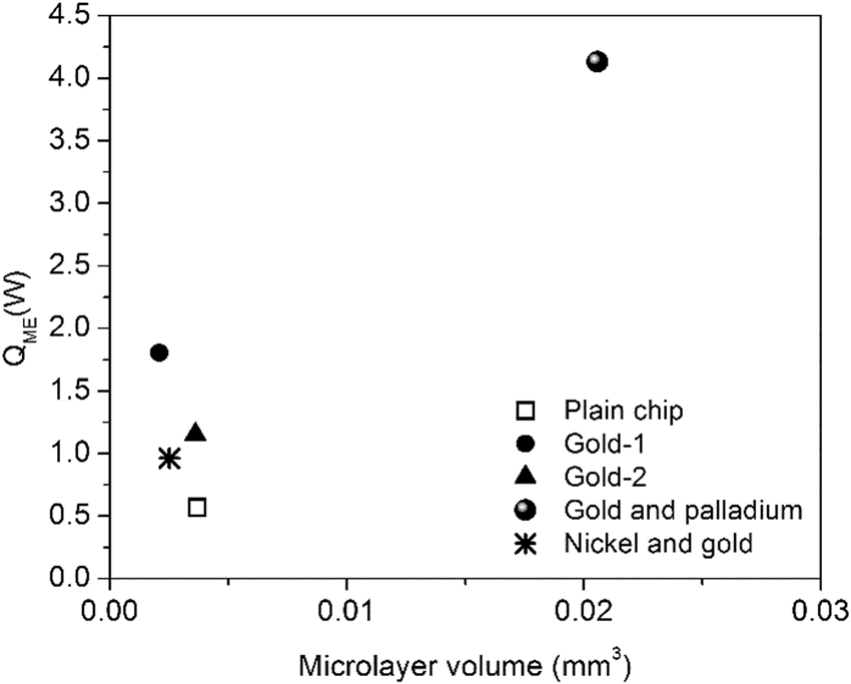
Microlayer volume and heat transfer from the microlayer under a bubble for the samples investigated here.

In analyzing the microlayer contribution, the maximum base diameter was used to calculate the volume of the microlayer film under the bubble. Utaka *et al*.^30^ conducted a series of experiments using interferometry technique to estimate the microlayer thickness. Using their work as guidance, the microlayer thickness based on the radial position from the nucleation site was estimated for each surface as listed in Table 3. A linear line fit on the experimental data by Utaka *et al*.^30^ was established in arriving at the microlayer thickness corresponding to the maximum bubble base diameter. The width span of this microlayer on the surface was assumed to be equal to the maximum base diameter attained on each surface (see Fig. 4). The microlayer heat transfer over the bubble cycle time was obtained using Eq. (3) as shown in Fig. 5. The microlayer heat transfer is analyzed as a function of the maximum base diameter and the bubble cycle time. The coupled motion of the CL increases the contribution from the microlayer by either increasing the bubble base diameter (gold and palladium) or by decreasing the bubble cycle time (gold-1, gold-2, and nickel and gold). Effectively, a high microlayer volume results in significantly higher evaporation rates as seen in the figure. A plain surface undergoing a stick-slip CL motion on the other hand increases the bubble cycle time and decreases the maximum diameter which cumulatively reduces the contribution from microlayer evaporation.

**Table 3:** Summary of heat transfer from microlayer and transient conduction mechanisms with CL velocity.

Surface	Microlayer thickness, µm	${\dot{{\boldsymbol{Q}}}}_{{\boldsymbol{ME}}}$ (W)	CL velocity (m/s)	t_adv_ (ms)	${\dot{{\boldsymbol{Q}}}}_{{\boldsymbol{TC}}}$ (W)
Plain chip	4.4	0.56	0.08	2	1.20
Gold and palladium	7.9	4.12	0.19	5.75	4.79
Gold-1	3.5	3.71	0.28	2	3.72
Gold-2	4.4	1.15	0.23	3.25	4.03
Nickel and gold	3.9	0.96	0.13	4.25	1.54

Transient conduction occurs as the CL sweeps over the heater surface during the advancing motion of the liquid. This motion creates the cold bulk liquid to quench the heater surface resulting in a cooling effect. The origin of this mechanism can be traced to Mikic *et al*.^14^ where the displaced volume of the bubble was assumed to be replaced by the liquid front instantaneously. Through advanced sensor based instrumentation techniques, Demiray and Kim^16^ demonstrated that the liquid front advanced gradually over the heater surface. The expression for surface heat flux per unit length can be estimated using,

4 $${\dot{Q}}_{TC}={\int }_{0}^{x}\frac{k{\rm{\Delta }}T}{\sqrt{\pi {\alpha }_{l}x}}dx$$

where, ${\dot{Q}}_{TC}$ is the heat transferred through transient conduction, *k* is the thermal conductivity, ${\alpha }_{l}$ is the thermal diffusivity, and *x* is the distance travelled by the CL.

Recent developments through controlled two-phase experimentation techniques^15,31^ suggest that the initial receding motion of the liquid increases the dry-spot region and drives the superheated liquid layer away from the dry-spot, thereby resulting in a lower heat transfer. On the other hand, it is shown that the advancing motion contributes efficiently by causing a gradual quenching of the region as the bubble departs which results in 3X heat transfer when compared to its receding motion^31^. Furthermore, a number of reports^15,16,29^ have suggested that the microlayer contributes ~15–25% of the total heat transfer and is expected to remain constant over the entire heat flux range. Therefore, we attribute a significant enhancement to occur through the transient conduction mechanism. Integrating Eq. (4) and transferring into time coordinates yields the following equation

5 $${\dot{Q}}_{TC}=\frac{k{\rm{\Delta }}T}{\sqrt{\pi {\alpha }_{l}}}v\sqrt{{t}_{adv.}}$$

The CL velocity *(v*) and the gradual wetting time *(t*_*adv*_ *)* is seen as critical parameters in explaining the degree of enhancement between the tested surfaces. Demiray and Kim^16^ established that the heat transfer varied as the square root of the time during the advancing liquid motion. The CL velocity was deduced from Fig. 4 during its advancing motion since the heat transfer during this period was shown to be significantly higher than its receding motion^31^. For a plain surface, the effective CL velocity is significantly reduced due to the pinning event. The chemically enhanced surfaces demonstrate a higher CL velocity. Table 3 summarizes the heat transfer obtained by solving Eq. (3) and Eq. (5).

## Results and Discussion

**Figure 6:**
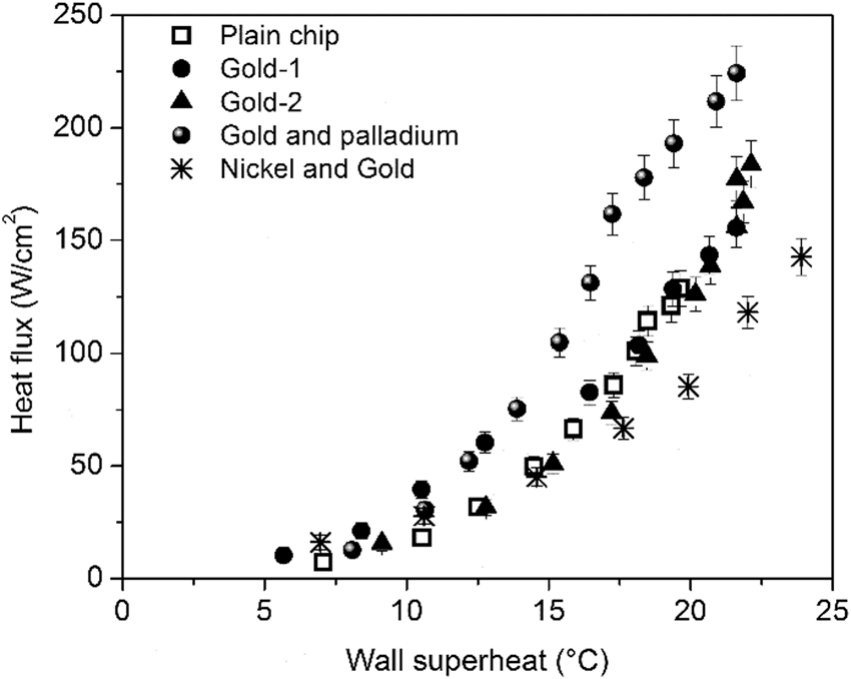
Pool boiling tests on the prepared samples with distilled water at atmospheric pressure.

Pool boiling experiments were conducted with degassed and distilled water at atmospheric pressure. Degassing was achieved by continuously boiling the liquid prior to conducting the boiling tests. Figure 6 shows the pool boiling curves obtained with heat flux on the Y-axis and wall superheat on the X-axis. As a baseline for enhancement comparisons, the plain surface was tested for its boiling performance. This surface reached a CHF of 128 W/cm^2^ at a wall superheat of 20 °C. A maximum CHF of 220 W/cm^2^ was obtained with the sputter coated sample comprising of gold and palladium. A CHF of 143 W/cm^2^ was obtained with the nickel and gold surface, while a CHF of 155 W/cm^2^ and 177 W/cm^2^ was obtained with the gold-1 and gold-2 surfaces, respectively. The wall superheats were similar for all the surfaces. An uncertainty analysis using the method of partial sums was conducted similar to Jaikumar and Kandlikar^27^ and is shown as error bars in the Fig. 6.

**Figure 7:**
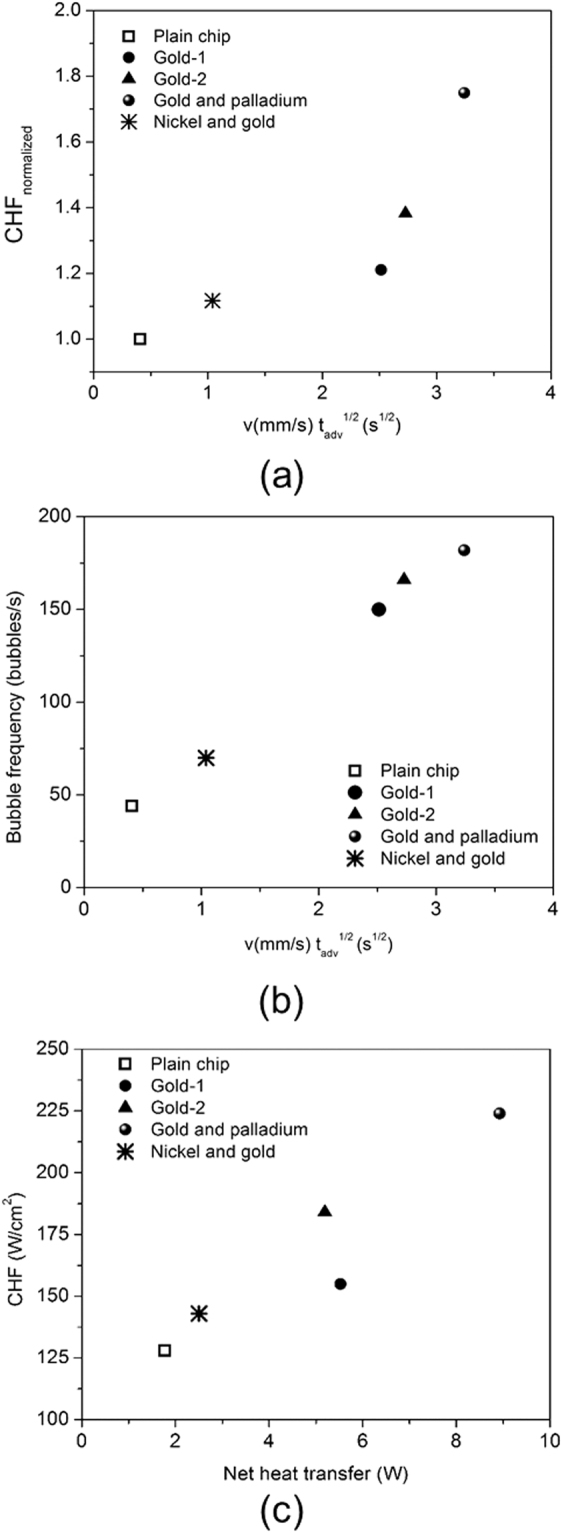
(**a**) Effect of transient conduction velocity and time on the normalized CHF enhancement. The CHF was normalized over the plain copper surface without chemical inhomogeneity. (**b**) Experimentally measured bubble frequency as a function of transient conduction parameters (CL velocity and advancing liquid front time). (**c**) Net heat transfer (microlayer evaporation and transient conduction) from a single bubble on the experimentally obtained CHF.

The CL velocity during its advancing motion is shown to be critical on surfaces at the nanoscale with enhanced coatings. Therefore, the product of CL velocity and gradual surface coverage time obtained previously is plotted against the normalized experimental CHF values as shown in Fig. 7a. The normalized CHF refers to the CHF on the chemically inhomogeneous surface divided by the CHF on a plain copper surface. This figure shows that an increase in the velocity is seen to increase the CHF. This leads to heat transfer enhancement by two important mechanisms: (a) the CL velocity affects the transient conduction contribution which is identified as the significant contributor for the enhanced performance characteristics on chemically enhanced nanoscale surfaces, and (b) lack of pinning events due to chemically induced inhomogeneity drives the CL motion on these surfaces through a change in the local dynamic contact angles (Fig. S3 in supplementary information). These attributes contribute to increased nucleation and rewetting characteristics on the surfaces. At higher heat fluxes, boiling becomes fully developed and large number of nucleation sites become active. Therefore the boiling characteristics is captured through the bubble frequency in the analysis to explain the CHF enhancement. Figure 7b shows the increase in bubble frequency as a function of the transient conduction parameters, namely CL velocity and liquid advancing time. A higher CL velocity results in the faster growth and departure of the bubble contributing to increased bubble frequency as seen in the figure.

The net heat transfer to each bubble from microlayer evaporation and transient conduction are plotted against the CHF values obtained experimentally as shown in Fig. 7c. The microlayer span is effective over the maximum base diameter that a bubble grows over on different surfaces. The pinning event experienced by the plain chip reduced the CL velocity which diminishes the effect from transient conduction mechanisms. The chemically enhanced surfaces experienced a continuous motion of the CL without any pinning events, and therefore these surfaces experienced a higher heat transfer from the transient conduction mechanisms. Figure 7a confirms the above observations showing that a chemically enhanced surface with higher CL velocity shows significantly higher CHF compared to the plain uncoated surface.

The analysis presented here supports a mechanism in which the CL moves smoothly over a chemically inhomogeneous surface. This effect is amplified when microscale features are absent, and therefore the surface chemistry effects are seen to drive the CL motion. The compositional change due to inhomogeneity induces a local wettability change along the CL in accordance with Tanner’s law.

## Conclusions

The motion of the CL was analyzed for surfaces developed with chemical inhomogeneities. Carefully designed experiments demonstrate the motion of the CL to occur in a coupled fashion on these surfaces which is shown to be influenced by the CL velocity. The coupled motion of CLs is recognized as an important mechanism for enhancement in boiling heat transfer. Therefore, a range of surfaces with different chemical compositions were created and their pool boiling performance characteristics was evaluated. An isolated bubble from nucleation to departure revealed that the plain chip suffered multiple pinning events during the ebullition cycle, resulting in degraded heat transfer performance. The surfaces developed through chemical inhomogeneities depicted a coupled motion of the CL resulting in higher heat transfer. Furthermore, the CL velocity was shown to correlate almost linearly with an increase in CHF and bubble frequency, thereby increasing the total contribution from the microlayer evaporation and transient conduction mechanisms when compared to a plain chip. This study shows that the enhancement mechanism at the nanoscale is affected by the compositional gradient to drive the CL over the surface. Therefore, designing nanoscale surfaces with compositional and wettability transitions will be beneficial in further improving the performance.

## Methods

### Copper surfaces

The copper test surfaces used in this study consisted of a 17 mm × 17 mm area with a central 10 mm × 10 mm boiling region. The test surfaces were prepared using an oxygen free Copper rod (supplied by: onlinemetals.com, #C101) with a purity of 99.99%. The thermal conductivity of this section was 391 W/m-K (±9 W/m-K). The copper rod was machined to required dimensions using a computer numerical control micro-milling process. A grinding operation was then performed to bring the roughness value to ~1 µm. The boiling surface was cleaned with IPA and distilled water. The bottom section comprised of a shaft which housed the thermocouple holes to measure the heat flux and surface temperature.

### Electrochemical and sputter coatings

A Casewell Plug and Plate® electroplating equipment was used in the experiments. This technique employs a stainless steel wick wand to transfer the solution uniformly over the surface. The aforementioned test section formed one of the electrodes, while the wick wand dipped in the desired solution formed the other electrode. A 4.5 V-DC was applied to initiate the plating process. A single stroke across the surface formed a coated layer. To generate uniform coatings with acceptable surface coverage, the number of strokes was increased. Gold-1 and Gold-2 surfaces comprised of 10 and 100 strokes, respectively. The nickel and gold surfaces were developed by mixing equal proportions (by vol.) of gold and nickel solutions and subsequently electroplating the surfaces under similar conditions.

### Experimental setup

An experimental setup similar to Jaikumar and Kandlikar^27^ was used in this study. The setup consisted of three main sections (a) ceramic test chip holder, (b) a glass water bath with clear visualization access, and (c) a heater block to supply the required heat to the test section. A detailed explanation of the test section assembly and test procedure is provided in the supporting information.

### Data availability

All data generated or analyzed during this study are included in this published article (and its Supplementary Information files).

## Electronic supplementary material

Supporting information
