# Supplementary material for: Coupled Motion of Contact Line on Nanoscale Chemically Heterogeneous Surfaces for Improved Bubble Dynamics in Boiling
Source: Sci Rep. 2017 Nov 16;7:15691. doi: 10.1038/s41598-017-16035-8 (PMC5691056; doi:10.1038/s41598-017-16035-8)
Supplement: Supplementary file 1 — Supporting information [file 41598_2017_16035_MOESM1_ESM.pdf]

# Coupled Motion of Contact Line on Nanoscale Chemically Heterogeneous Surfaces for Improved Bubble Dynamics in Boiling

*Arvind Jaikumar[1], Satish G. Kandlikar[1,2*]*

[1]Microsystems Engineering, Rochester Institute of Technology, 76 Lomb Memorial Drive, Rochester, NY 14623
[2]Mechanical Engineering, Rochester Institute of Technology, 76 Lomb Memorial Drive, Rochester, NY 14623

**Corresponding Author**

*Satish G. Kandlikar

sgkeme@rit.edu,

Mechanical Engineering, Rochester Institute of Technology, 76 Lomb Memorial Dr., Rochester,

NY, 14623, U.S.A.

Telephone: +1-(585)-475-6728

Fax: +1-(585)-475-6879

# Supporting information

**High speed images of bubble diameter on chemically enhanced surfaces:**

Figure S1 shows the bubble image sequence obtained for the gold and palladium surface at 4000 fps using a Photron fastcam. This surface is characteristic to smooth and continuous motion (coupled) of the contact line.  Similar time steps to Fig. 1 (in the manuscript) for a plain chip is used here to enable easy comparison of the contact line movements. Primary observation from Fig. S1 is the lack of pinning events on the surface. Consequently, the contact line moves faster on this surface. The base contact line diameters are also significantly higher on this surface. The large base diameters increase the contribution from microlayer evaporation.

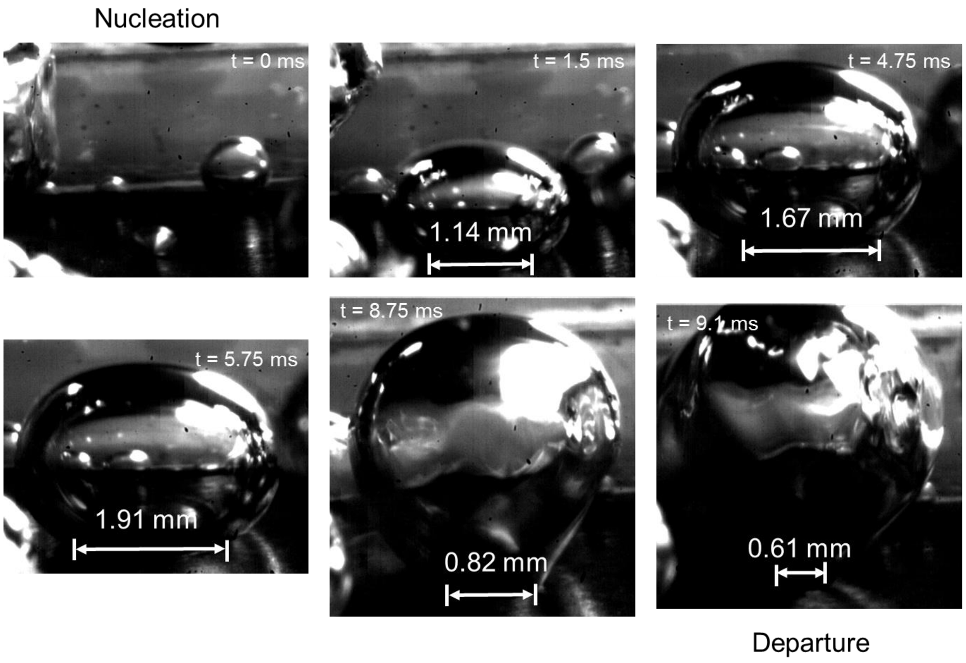


Figure S1. High speed image sequence of bubbles nucleating on the chemically enhanced surface.

**Multiple bubble data:**

The figure S2 shows the bubble base diameters measured for multiple isolated bubbles on each surface. The plain chip S2a shows pinning and moving events on all the bubbles. Bubble 4 exhibits slightly higher base diameter values which was attributed to the local fluid temperature and heat flux levels. The chemically enhanced surface S2b-d show a continuous and smooth coupled motion of the contact line.

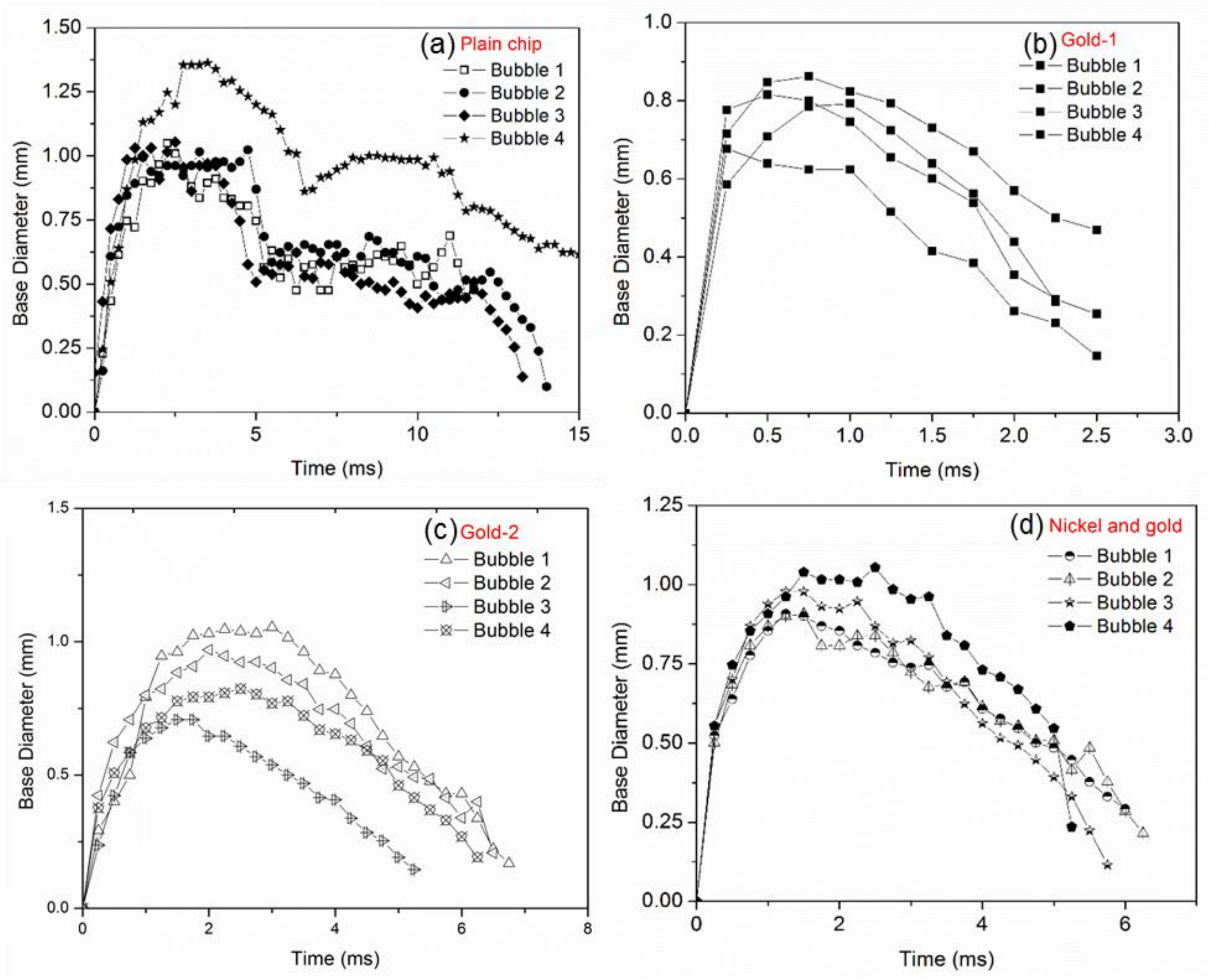


Figure S2. Base radius diameter from nucleation to departure for (a) plain chip, (b) Gold-1, (c) Gold-2, and (d) Nickel and gold.

## Effect of Dynamic Contact Angles

Figure S3 shows the change in dynamic contact angles (CA) for a bubble growing on a plain copper chip and a chemically inhomogeneous gold and palladium surface in a saturated pool of water. The plain copper chip shows a minimal change in the wettability characterized by the dynamic CA during the initial receding and subsequent pinning durations on the surface. A change in the dynamic contact angle from 45° to 63° in the first advancing motion and 63° to 82° in the second advancing motion was observed. The gold and palladium surface demonstrated a highly pronounced wettability change during both the advancing and receding motion. These results suggested that the CL motion was driven by the change in the dynamic contact angles as described by Tanners law.

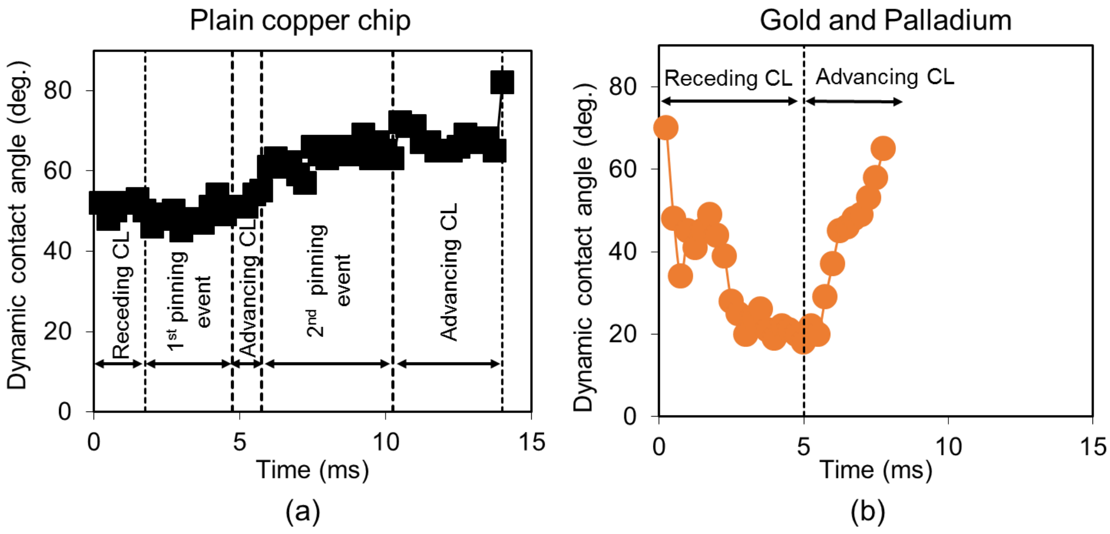


Figure S3. Effect of dynamic contact angles during the bubble growth for (a) plain copper chip, (b) gold and palladium surfaces.

**Pool boiling experimental setup**

Figure S4 shows a schematic of the pool boiling test setup used in this study. The main components of the test setup included a test chip, a water bath and a heating unit. The test chip was housed in a ceramic chip holder held on the bottom garolite plate over which a quartz glass water bath measuring 14 mm × 14 mm × 38 mm was assembled. A rubber gasket was used to seal the two contacting surfaces and it also covered the area outside the 100 $mm^2$ boiling surface. Additionally, this area was further covered with a Kapton® tape to prevent it from participating in heat transfer. A middle garolite plate held the water bath on the upper side and was connected to the top aluminum plate by means of two stainless steel socket head cap screws. A water reservoir was mounted between the middle garolite plate and top aluminum plate to replenish water in the glass water bath. The water reservoir was sealed with rubber gaskets on all sides to ensure against leakage. The top aluminum plate was provided with two circular openings for the saturation thermocouple probe and a 60-VDC, 200 W auxiliary cartridge heater to maintain water in the reservoir at saturation by boiling it continuously.

The bottom section of the setup consisted of four 120-VDC, 200 W capacity cartridge heaters inserted into a copper heater block. The block consisted of a truncated portion measuring 10 mm × 10 mm × 40 mm that fits into the groove on the bottom side of the ceramic chip holder. This ensured that 10 mm × 10 mm surface of the heater is in contact with the test chip which also has a base section measuring 10 mm × 10 mm which facilitated 1D conduction from the heater to the test chip. Thus no heat spreader effect was utilized. Additionally, the copper block was housed on a ceramic sleeve to minimize heat losses. Four compression springs supported the bottom aluminum plate that provided the required degree of movement to establish contact between the test chip and the heater

block and accommodate for any thermal expansion during testing. A shaft pin connected the bottom garolite plate and the work desk which ensured stability of the setup during testing.

A National Instruments cDaq-9172 data acquisition system with NI-9213 temperature module was used to record the temperature. A LabVIEWVR virtual instrument displayed and calculated the surface temperature and heat flux.

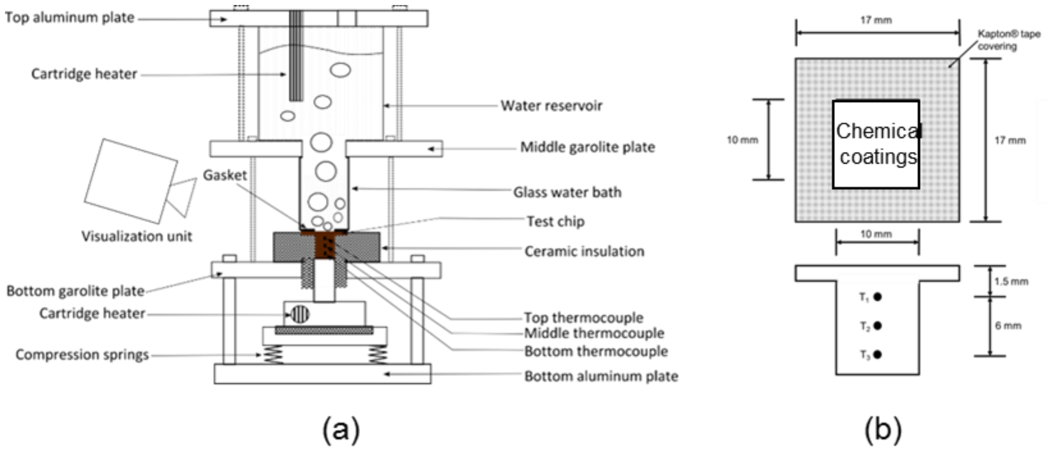


Figure S4. Schematic of (a) Experimental pool boiling test setup, (b) test chip.

## Test chip

The test section used in this study was a 10 mm × 10 mm boiling surface in the middle of a square 17 mm square thin copper chips as shown in Fig. S4b. The heater side consisted of a 10 mm × 10 mm × 9 mm protrusion with three 0.76 mm holes drilled 3 mm apart to accommodate the thermocouples. The effect of contact resistance in the heat flux and surface temperature calculation was eliminated by holes drilled in the test chip. Three K-type thermocouples are inserted into these holes to read temperatures $T_1$, $T_2$ and $T_3$.
